# Supplementary material for: Characteristics of Electroencephalogram in the Prefrontal Cortex during Deep Brain Stimulation of Subthalamic Nucleus in Parkinson’s Disease under Propofol General Anesthesia
Source: Brain Sci. 2022 Dec 29;13(1):62. doi: 10.3390/brainsci13010062 (PMC9856588; doi:10.3390/brainsci13010062)
Supplement: Supplementary file 1 [file brainsci-13-00062-s001.zip › brainsci-2087833-supplementary.pdf]

Table S1 Comparison of EEG parameters between two groups of patients with Parkinson's disease in three states

	Total	No benzodiazepine users	Benzodiazepine users	*p* value
	n=43	n=25	n=18	
Awake state before anesthesia				
PSI	89.86±6.89	88.90±8.42	91.19±3.74	0.92[b]
SEF_L, Hz	22.81±4.14	22.43±4.36	23.34±3.89	0.49[a]
SEF_R, Hz	22.58±4.13	21.66±4.23	23.87±3.73	0.08[a]
Total time of SUP, min	0.35 (0, 6.5)	0.43 (0, 6.5)	0.13 (0, 5.48)	0.26[b]
Maximum SUP ratio, %	2 (0, 28)	2 (0, 8)	2 (0, 28)	0.62[b]
EMG, %	59.90±22.00	56.20±23.58	65.04±19.04	0.20[a]
During propofol anesthesia				
PSI	48.68±12.65	49.33±13.31	47.79±12.00	0.60[b]
SEF_L, Hz	12.70±3.33	12.20±3.66	13.40±2.74	0.25[a]
SEF_R, Hz	12.61±3.29	12.18±3.63	13.21±2.74	0.32[a]
Total time of SUP, min	1.2(0, 124.10)	2.55(0, 124.10)	1.02(0, 29.17)	0.77[b]
Maximum SUP ratio, %	6 (0, 34)	4 (0, 34)	6 (0, 28)	0.98[b]
MER period				
PSI	62.46±13.07	60.94±12.76	64.58±13.58	0.20[b]
SEF_L, Hz	15.42±4.07	14.54±3.96	16.65±4.01	0.10[a]
SEF_R, Hz	15.36±4.07	14.51±4.02	16.55±3.94	0.11[a]
Total time of SUP, min	2.57(0, 141.33)	4.83(0.35, 141.33)	1.40 (0, 12.40)	0.01[b]
Maximum SUP ratio, %	6 (0, 30)	6(4, 30)	4 (0, 14)	0.02[b]

Numbers indicate means ± standard deviations (SD) or median (minimum, maximum). Differences between the two groups were analyzed using the t testa or Wilcoxon rank sum testb. Signifificance was recognized when p <0.05. EEG:electroencephalogram, PSI: Patient State Index, SEF_L: spectral edge frequency, left side, SEF_R: spectral edge frequency, right side, SUP: Suppression, EMG: electromyography, MER: microelectrode recording.

Table S2 Comparison of average power spectral density at different frequencies between two groups of patients with Parkinson's disease in three states

	No benzodiazepine users n=25	Benzodiazepine users n=18	*p* value
Awake state before anesthesia			
SWO	8.86 (3.54)	9.09 (6.57)	0.86[b]
Delta	5.95 (3.46)	4.63 (5.38)	0.68[b]
Theta	-2.85 (3.69)	-3.56 (3.51)	0.30[b]
Alpha	-5.08 ±2.19	-4.84 ±2.67	0.75[a]
Beta	-5.46 ±3.82	-4.92 ±3.92	0.66[a]
Gamma	-5.79 ±5.27	-4.75 ±4.78	0.51[a]
Propofol anesthesia state			
SWO	10.24 ±2.46	10.16 ±2.06	0.90[a]
Delta	6.66 ±2.06	6.65 ±1.89	0.98[a]
Theta	-2.53 (2.64)	-1.73 (4.52)	0.33[b]
Alpha	-3.47 ±2.76	-2.18 ±3.59	0.19[a]
Beta	-11.01 ±2.99	-9.48 ±3.51	0.13[a]
Gamma	-17.40 ±2.04	-16.36 ±2.64	0.15[a]
MER period			
SWO	4.64 ±3.10	5.21 ±3.11	0.55[a]
Delta	2.48 (3.45)	2.39 (2.46)	0.96[b]
Theta	-4.57 ±2.13	-3.96 ±1.98	0.35[a]
Alpha	-5.77 (3.18)	-5.12 (5.96)	0.18[b]
Beta	-13.20 ±2.65	-10.92 ±3.78	0.03[a]
Gamma	-18.28 ±2.38	-16.24 ±3.37	0.03[a]

Numbers indicate means ± standard deviations or median (interquartile range). Differences between the two groups were analyzed using the *t* test[a] or Mann-Whitney U test[b]. Signifificance was recognized when $p < 0.05$. SWO: slow-wave oscillation, MER: microelectrode recording.
